# The Inactivation of Arabidopsis *UBC22* Results in Abnormal Chromosome Segregation in Female Meiosis, but Not in Male Meiosis

**DOI:** 10.3390/plants10112418

**Published:** 2021-11-09

**Authors:** Ling Cao, Sheng Wang, Lihua Zhao, Yuan Qin, Hong Wang, Yan Cheng

**Affiliations:** 1 State Key Laboratory of Ecological Pest Control for Fujian and Taiwan Crops, Center for Genomics and Biotechnology, College of Plant Protection, Fujian Agriculture and Forestry University, Fuzhou 350002, China; lic792@mail.usask.ca (L.C.); lihua.zhao@slu.se (L.Z.); yuanqin001@foxmail.com (Y.Q.); 2 Department of Biochemistry, Microbiology and Immunology, University of Saskatchewan, Saskatoon, SK S7N 5E5, Canada; sheng.wang@usask.ca

**Keywords:** Arabidopsis, chromosome segregation, DMC1, female meiosis, K11-linked, megaspore mother cell, ubiquitin-conjugating enzyme, UBC22

## Abstract

Protein ubiquitination is important for the regulation of meiosis in eukaryotes, including plants. However, little is known about the involvement of E2 ubiquitin-conjugating enzymes in plant meiosis. Arabidopsis UBC22 is a unique E2 enzyme, able to catalyze the formation of ubiquitin dimers through lysine 11 (K11). Previous work has shown that *ubc22* mutants are defective in megasporogenesis, with most ovules having no or abnormally functioning megaspores; furthermore, some mutant plants show distinct phenotypes in vegetative growth. In this study, we showed that chromosome segregation and callose deposition were abnormal in mutant female meiosis while male meiosis was not affected. The meiotic recombinase DMC1, required for homologous chromosome recombination, showed a dispersed distribution in mutant female meiocytes compared to the presence of strong foci in WT female meiocytes. Based on an analysis of F1 plants produced from crosses using a mutant as the female parent, about 24% of female mutant gametes had an abnormal content of DNA, resulting in frequent aneuploids among the mutant plants. These results show that UBC22 is critical for normal chromosome segregation in female meiosis but not for male meiosis, and they provide important leads for studying the role of UBC22 and K11-linked ubiquitination.

## 1. Introduction

Meiosis is a modified cell cycle with one round of DNA replication and two rounds of cell division, producing haploid gametes from diploid somatic cells. In plants, a number of genes with a possible role in meiosis have been identified, but many aspects regarding how the entry and progression of meiosis are controlled remain unknown [1]. 

Many events specific to meiosis occur in meiosis I. One essential aspect is the pairing of homologous chromosomes. The specific mechanisms for the initial recognition and pairing of homologous chromosomes are still not fully understood and they may vary depending on species [2,3]. The paired homologous chromosomes are held together by the synaptonemal complex (SC) and meiotic cohesion complex (cohesin). The SC is a highly conserved structure formed in prophase I along the length of homologous chromosomes. It consists of a pair of parallel strands, called lateral elements, with the loops of sister chromatids attached, and the lateral elements are linked by a central element [2]. In plants, two axis-associated proteins have been identified: Arabidopsis ASY1 and ASY3, and the rice homologs PAIR2 and PAIR3 [4,5,6,7]. In the rice *PAIR2* and *PAIR3* mutants, homologous chromosomes fail to undergo synapsis and as a result appear as unpaired univalents later in prophase I [5,6]. Additionally, the inactivation of Arabidopsis *ASY1* or *ASY3* results in the failure of synapsis and a disruption in the formation of the SC [7,8]. These results show that the two plant axial proteins play an important role in the pairing and synapsis of homologous chromosomes.

The meiotic cohesin consists of four highly conserved proteins (SMC1, SMC3, REC8 and SCC3) [9]. In meiosis I, cohesin on the sister chromatid arm is removed by separase, or separin, which abolishes the linkage of homologous chromosomes [10,11], while centromeric cohesion is maintained until meiosis II to ensure that the sister chromatids are not separated before meiosis II [12,13,14]. It has been found in *Drosophila* that the assembly of the SC depends on two meiosis-specific cohesion complexes, with one complex required for interhomolog interactions and the other required for sister chromatid interactions [15]. In Arabidopsis, the mutation of separase (ESP) results in a mixture of fragmented chromosomes and intact bivalents in the meiosis of micromeiocytes [16].

Another crucial aspect of meiosis is the recombination between the homologous chromosomes [1,17]. At the initiation stage of meiotic recombination, DNA double-strand breaks (DSBs) are formed, in which the evolutionarily conserved topoisomerase-like enzyme SPO11 plays a critical role [18,19]. The DSBs need to be repaired before the segregation of homologous chromosomes [20]. 

Two recombinases and eukaryotic homologues of the *E. coli* recA, DMC1 (disrupted meiosis cDNA1) and RAD51, function in DSB repair and recombination [21]. Based on the results from the protist *Tetrahymena*, it has been proposed that RAD51 functions in intrachromosomal recombination, whereas DMC1 is required for interhomolog recombination [20]. In Arabidopsis, both the *dmc1* and *rad51* mutants had normal vegetative growth, but meiosis in male and female meiocytes was disturbed, indicating that they are critical for meiosis but dispensable for mitosis [22,23]. In the *atdmc1* mutant, the chromosomes failed to synapse and generally appeared as ten fully condensed univalents [22]. In the *atrad51* mutant, the chromosomes also failed to synapse and in addition were fragmented [23]. Results have indicated that Arabidopsis DMC1 and RAD51 are localized at the opposite ends of a meiotic DSB, suggesting that they have different functions [24]. Further, results from the complementation of Arabidopsis single, *rad51*, and double, *dmc1* and *rad51*, mutants with RAD51-GFP have suggested that DMC1 plays a major role, with RAD51 having a support role in meiotic recombination in flowering plants [25]. 

Protein ubiquitination is essential for the regulation of the cell cycle, with many proteins being potential targets of modification [26,27]. The machinery consists of a three-enzyme cascade, with the ubiquitin-activating (E1), ubiquitin-conjugating (E2) and ubiquitin (Ub) ligase (E3) enzymes operating to attach a single ubiquitin (monoubiquitin) or a chain of ubiquitin (polyubiquitin) onto the substrate proteins. Protein ubiquitination serves various functions, such as protein recognition and degradation, cellular localization and the modulation of protein activity and protein interactions [28,29]. The polyubiquitin chains can be formed through one of the seven lysine residues of Ub, and polyubiquitin chains linked through different lysine residues can provide signals for various functions [30]. Lysine 48 (K48)-linked polyubiquitination, the most common type of polyubiquitination, is generally known to target substrates to the 26S proteasome for degradation [30,31]. The noncanonical K11-linked chains also act as targeting signals for proteasome degradation, but are generally associated with regulation of the cell cycle [32,33,34].

Protein ubiquitination and in particular two E3 ligase complexes, the anaphase promoting complex/cyclosome (APC/C) and Skp1-cullin-F-box (SCF) complexes, also play important roles in plant meiosis [35,36]. Arabidopsis APC/C is composed of at least 14 core subunits [36]. The Arabidopsis APC/C core subunit APC8 has been shown to be important for male meiosis, since a point mutant has several defects in male meiosis, but interestingly not in female meiosis [37]. Arabidopsis TDM1 (three division mutant 1) is supposedly a putative meiotic APC/C component, and can interact with the APC/C subunit APC3b and co-activator CDC20.1 [38]. TDM1 functions to ensure correct meiotic termination at the end of meiosis II, and its mutation leads to an abnormal third division. Limited research results have also shown the importance of the SCF complex in plant meiosis. For instance, the Arabidopsis SKP1-LIKE1 (ASK1) protein, as a key component of the SCF complex, controls homologue separation in male meiosis [39].

E2 enzymes are mainly responsible for the assembly and topology of Ub chains, which determine the consequence of ubiquitination for the modified proteins [40,41]. Although functions have been demonstrated for E2 enzymes in many processes of plants [42], essentially no information is available about the role of any specific E2 enzyme in plant meiosis. 

Arabidopsis UBC22 is the sole member in one of the 14 subfamilies of E2 enzymes [43]. Its similarity to E2 enzymes in animals and humans, responsible for K11-linked ubiquitination and the ability to catalyze the formation of K11-linked Ub dimers in vitro, suggest that UBC22 represents a unique subfamily of plant E2 enzymes responsible for K11-linked ubiquitination [44]. The most prominent phenotypes of Arabidopsis *ubc22* mutants lie in female reproductive development, including the absence of or abnormally functioning megaspores (FMs), abnormal embryo sacs and aborted ovules [44]. In addition, a range of phenotypic changes during vegetative development were reported more recently [45]. Here, we further investigated the role of UBC22 in meiosis and report that the *ubc22* mutant plants displayed abnormalities in chromosome behavior and DMC1 protein distribution in the meiosis of female meiocytes, but not in male meiocytes. There was a frequent occurrence of aneuploids in *ubc22* mutant plants. These results indicate a critical role of UBC22 in plant female meiosis.

## 2. Results

### 2.1. Analysis of Megasporogenesis and Female Meiosis Using Callose Staining

**Figure 1:**
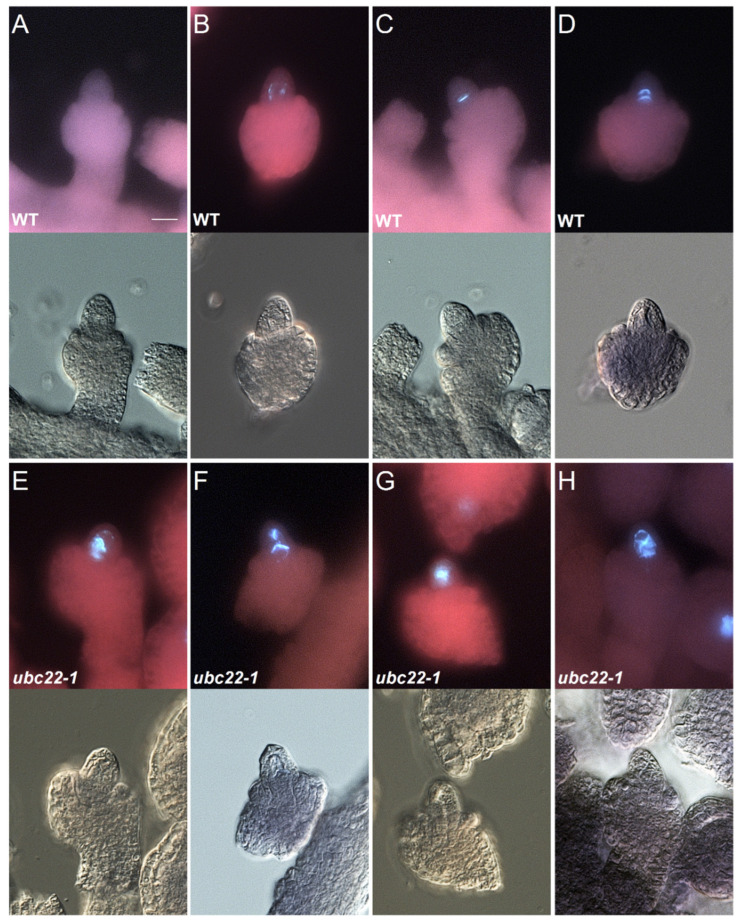
Callose formation in the early stages of megasporogenesis in WT and *ubc22-1* mutant ovules. For each ovule, the callose fluorescent image is shown on the top, and the bright-field image is shown on the bottom. WT (**A**–**D**) and *ubc22-1* mutant (**E**–**H**). Scale bar in (A): 10 μm. (**A**) No callose staining in a WT ovule before meiosis. (**B**) Deposition of callose along the cell wall of a WT MMC. (**C**) A clear callose band indicating the place of newly formed cell plate from the first meiotic division by a WT MMC. (**D**) Two clear callose bands as a result of the first meiotic division and the second division by the nucleus close to the chalazal end. (**E**) A callose band with diffused callose disposition in a *ubc22-1* MMC. (**F**) A callose band as well as callose deposition in other locations of a *ubc22-1* MMC. (**G**) Strong callose staining in the center of a *ubc22-1* MMC, but the callose band is not clear. (**H**) Disorganized callose deposition without a clear band in a *ubc22-1* MMC.

We previously observed that the majority of *ubc22* mutant embryo sacs displayed severe defects and often contained no gamete nuclei, and that the FM was either absent or abnormal in over 70% of mutant ovules [44]. To investigate more specifically the effect of *UBC22* inactivation on megasporogenesis, we used callose as a marker with which to examine female meiosis. Callose, a polysaccharide composed of glucose residues linked through β-1,3-linkages, is present at the cell plate during meiosis prior to cytokinesis, and has been used as a convenient cytological marker for meiosis in megaspore mother cells (MMCs or female meiocytes) [46]. The presence of callose can be visualized following aniline blue staining. In a WT MMC, initially no callose was observed in the ovule (Figure 1A), and just prior to meiosis there was some callose deposited along the cell wall (Figure 1B). After the first meiotic division, the fluorescence signal became concentrated at the site of the cell plate, appearing as a band between the daughter cells of meiosis I (Figure 1C). Following the second meiotic division, an extra callose band beneath the first callose band appeared, as a result of the division by the nucleus close to the chalazal end (Figure 1D). Thus, based on the callose staining pattern, we could infer approximately the stages of an MMC during meiosis in WT ovules.

In the *ubc22* mutant, the callose deposition often displayed abnormalities (Figure 1E–H). We surveyed callose deposition patterns during the early stages of meiosis in the WT and mutant ovules using floral buds at similar stages (with the inner integument just emerging to below half the length of the nucellus). In the WT, about 26% of ovules showed weak callose staining along the meiocyte cell wall (Figure 1B), indicating that the MMC was about to enter meiosis, while 74% of the WT ovules had clear callose band(s) (Table 1). Among the 74% ovules with clear callose bands, 28.2% and 46.2% of ovules showed one and two clear callose bands, respectively (Figure 1C,D). In contrast, only 10.7% of the mutant ovules showed one or two clear callose bands. About 17.5% of the mutant ovules had a callose band, but also had strong callose staining in other locations or an odd pattern (Figure 1E–G, Table 1). A large portion (36.9%) of the mutant ovules did not have a clear callose band, and instead showed much disorganized and diffused callose deposition (Figure 1H, Table 1).

**Figure 2:**
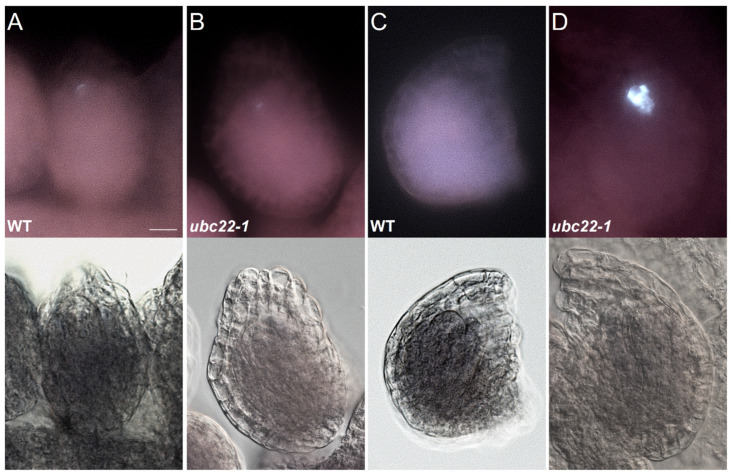
Presence of callose in the late stages of megasporogenesis in WT and *ubc22-1* mutant ovules. For each ovule, the callose fluorescent image is shown on the top and the bright-field image is shown on the bottom. WT (**A**,**C**) and *ubc22-1* mutant (**B**,**D**). Scale bar: 10 μm. (**A**) Weak callose staining at the micropylar end in a WT ovule after meiosis. (**B**) Weak callose staining at the micropylar end in a *ubc22-1* mutant ovule after meiosis. (**C**) No callose signal in a WT ovule. (**D**) Strong disorganized callose staining in a *ubc22-1* ovule.

After meiosis, there was a weak fluorescence signal at the micropylar end, where the degenerating megaspores were located in the WT ovules (Figure 2A), and a similar pattern was observed in some mutant ovules (Figure 2B). Then, the callose staining gradually disappeared in the WT ovules (Figure 2C). A survey of WT ovules (from the fourth bud counting from the newly opened flower) revealed that 1.6% of them still showed two bands of callose, 51.6% had some callose staining at the micropylar end and 46.8% did not show any callose staining (Table S1). For *ubc22* mutant ovules at the same developmental stage, 0.8% had two bands of callose, 29.8% had callose staining at the micropylar end and 6.9% did not show any callose staining (Table S1). In addition, 62.6% of the mutant ovules showed disorganized and strong callose staining (Figure 2D and Table S1). These results suggest that abnormality during female megasporogenesis in *ubc22* mutants likely starts during the first meiotic division and continues into the later stages of megasporogenesis.

### 2.2. Analysis of Meiosis in Female and Male Meiocytes Using Chromosome Spreading

**Figure 3:**
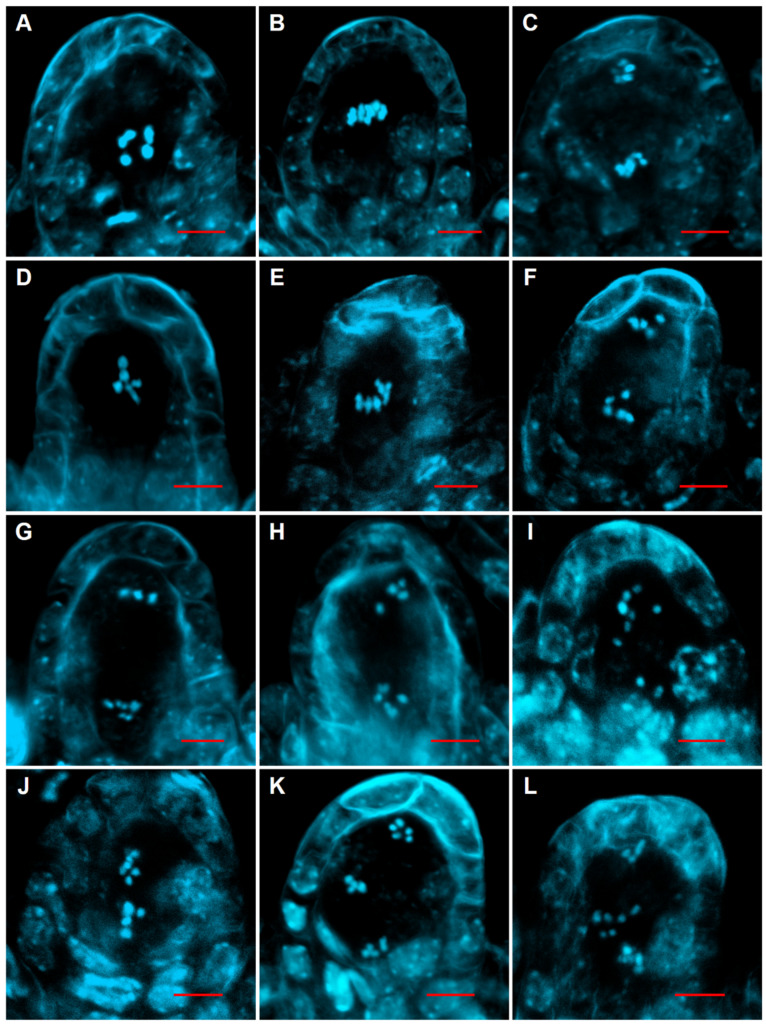
Female meiosis in the WT and *ubc22* mutant. The ovules of the WT (**A**–**C,K**) and diploid *ubc22-1* mutant (**D**–**J,L**) were dissected, processed and stained with DAPI and Calcofluor White. Meiotic chromosomes were observed with a confocal laser scanning microscope. (**A**,**D**) Prophase I (diakinesis) in the WT (**A**) and mutant (**D**), showing five brightly condensed bivalents. (**B**,**E**) The five bivalents were organized on the metaphase I plate in the WT (**B**) and mutant (**E**). (**C**,**F**) Telophase I in the WT (**C**) and mutant (**F**), showing sets of evenly segregated chromosomes located at the two poles. (**G**) Uneven segregation of homologous chromosomes with three chromosomes on the top and seven at the bottom. (**H**) Uneven segregation of homologous chromosomes with four on the top. (**I**) Uneven segregation of homologous chromosomes with seven on the top. (**J**) Uneven segregation of homologous chromosomes with six on the top. (**K**) Meiosis II in WT. (**L**) Meiosis II in the mutant, showing uneven chromosome segregation. Scale bars = 5 μm.

Next, we characterized chromosome behavior during female meiosis of *ubc22* mutants compared with the WT using the chromosome spreading technique. For this analysis, we used *ubc22* mutant plants which had been determined to be diploids by flow cytometry, since some *ubc22* mutant plants may have an altered content of nuclear DNA (see next section). The ovules were processed, stained with DAPI and Calcofluor White and observed using confocal microscopy. In the WT ovules, the paired and condensed homologous chromosomes were typically observed at prophase I and metaphase I (Figure 3A,B). At late anaphase or telophase I, five chromosomes could also be observed in each of the two daughter cells (Figure 3C). In the *ubc22* ovules, the homologous chromosomes paired normally and appeared as bivalents in prophase I and metaphase I (Figure 3D,E). In some *ubc22* ovules, the homologous chromosomes were segregated evenly to the opposite poles at late anaphase I (Figure 3F). However, it was frequently observed in other *ubc22* ovules that the homologous chromosomes were segregated unevenly, with variable numbers of chromosomes at the two poles (Figure 3G–J). After meiosis I, the MMC in WT proceeded quickly into the second meiotic division, which is similar to mitosis, involving the segregation of sister chromatids and usually producing four haploid megaspores. In the WT, the separation of five chromosomes was observed in the two daughter haploid cells, although the divisions of the two haploid cells from the first division might not be synchronous (Figure 3K). For the *ubc22* mutant, although uneven chromosome numbers in the two daughter cells derived from the first division were also observed (Figure 3L), uneven segregation of the chromatids in the second division was rarely observed. Nevertheless, the aberrant chromosomal segregation observed suggests that female meiosis in the *ubc22* mutant is disturbed, starting from meiotic division I, resulting in frequent uneven chromosome segregation.

We also examined chromosome behavior in male meiocytes, and the results showed that male meiosis was normal (Figure S1), consistent with previous findings that suggested that the pollen grains of *ubc22* mutants were mostly normal with three nuclei [44].

### 2.3. Analysis of MMC Meiosis Using DMC1 Immunostaining

**Figure 4:**
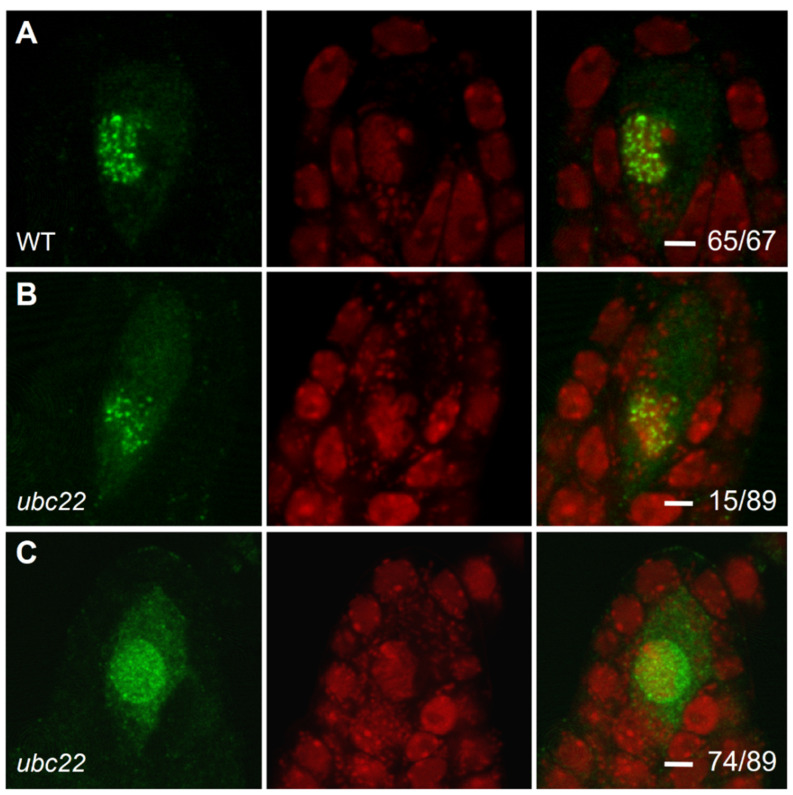
DMC1 immunolocalization in WT and *ubc22* mutant ovules. WT and *ubc22-1* mutant ovules were dissected, fixed and processed for immunostaining with an antibody against DMC1 (green color). They were also stained with propidium iodide (PI, red color). (**A**) In the WT ovules, DMC1 accumulates during prophase I in the MMC as foci along chromosome axes. (**B**) A small portion (about 17%) of the mutant ovules had a DMC expression pattern similar to the WT, although the foci were less intense. (**C**) In the majority of *ubc22-1* ovules (83%), DMC1 signals were spread out loosely in the nucleus. In each row, the left image shows DMC1 staining, the center shows PI staining and the right shows the overlay of the two images. The numbers in the right images indicate the frequencies of the ovules with their expression pattern. Scale bars = 2 µM.

The DMC1 (disrupted meiotic cDNA 1) protein is a meiosis-specific recombinase required for homologous chromosome recombination [22,25]. It is specifically expressed in meiosis and accumulated in prophase I to form many foci [24,47]. Thus, we performed ovule immunostaining using an antibody against DMC1. In the WT ovule, DMC1 was specifically expressed and accumulated as strong foci in the MMC in prophase I (Figure 4A and Figure S2A). In about 17% of the *ubc22* mutant ovules, DMC1 displayed a similar pattern, although the foci were less intense and more spread out in the nucleus (Figure 4B). In the majority (83%, or 73 out of 89) of the mutant ovules the foci were visibly weaker, and DMC1 also showed a more diffused distribution in the nucleus (Figure 4C and Figure S2B). These results show that the DMC1 protein was distributed much more evenly in the nucleus, and that its loading onto the foci might be affected in the mutant. 

ASY1 is a chromosome axis-associated protein important for homologous pairing and synapsis [4,5]. We further analyzed ASY1 localization using immunostaining. The results showed that ASY1 distribution was similar in the MMCs of the WT and *ubc22* mutants (Figure S3). 

### 2.4. Analysis of Nuclear DNA Content in F1 Plants from Reciprocal Crosses between the WT and ubc22-1 Mutant

**Figure 5:**
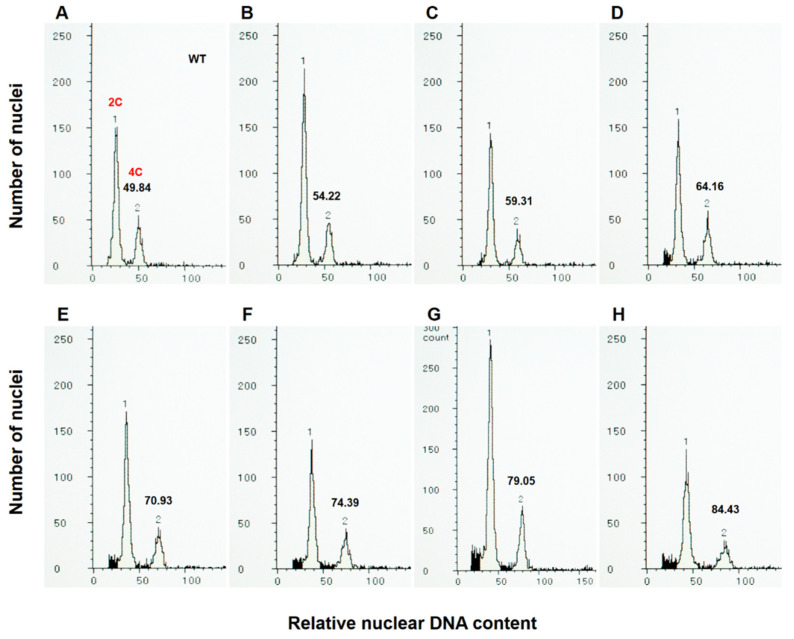
Histograms of flow cytometric analysis on the F1 plants from the cross between the WT and *ubc22-1* mutant with the WT as the paternal parent. Flow cytometry was performed using floral bud tissues of individual plants, and nuclei were stained with DAPI. The parameters of the flow cytometry were set so that the average WT 4C peak value was about 50. (**A**) Histogram of a control WT plant, showing two major peaks that represent nuclei with 2C and 4C DNA contents. (**B**–**H**) Histograms of F1 plants with various 4C values higher than 50, indicating that the plants were not diploids. Note that the DNA content in (**F**) is 1.5 times that in the WT (**A**), indicating that the plant had a nuclear DNA content of a triploid, while the other F1 plants were aneuploids.

To determine the impact of chromosome abnormalities in the *ubc22* mutants on the ploidy level, in addition to whether female or male meiosis is affected, we analyzed the nuclear DNA content in the F1 plants produced from reciprocal crosses between the WT and *ubc22-1* mutant. For the flow cytometric analysis, we used young floral bud tissues, since they produced only two major peaks, representing 2C and 4C nuclei (Figure 5A), while the leaf tissues had extra peaks representing 8C and 16C nuclei [48,49]. We adjusted the flow cytometric settings so that the values for the 4C peaks from the WT floral tissues were about 50 (Figure 5A), which allows different plants to be conveniently compared based on the values of the 4C peaks. All the WT plants and F1 plants from the cross with the WT as the maternal parent had a 4C value close to 50 and within the range of 47.5 to 52.5. On the other hand, it was observed that many F1 plants from the cross with the *ubc22* mutant as the maternal parent had a nuclear DNA content higher than the diploid (Figure 5B–H), including aneuploids (Figure 5B–E,G,H) and a triploid (Figure 5F). 

**Figure 6:**
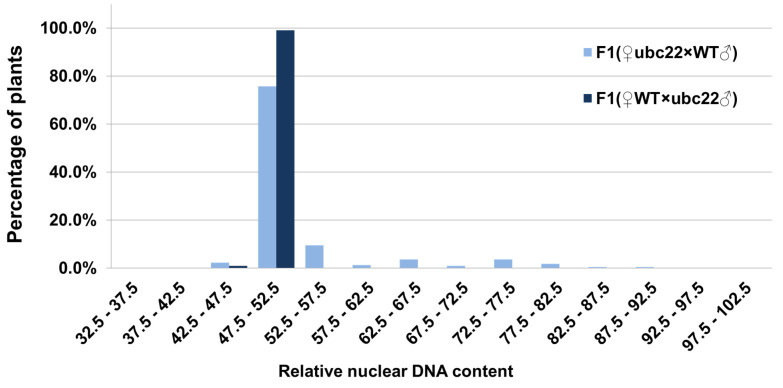
Relative nuclear DNA contents in F1 plants from the reciprocal crosses between the *ubc22-1* mutant and WT. F1 plants produced from reciprocal crosses between the *ubc22* mutant and WT were grown and their floral buds were used for flow cytometric analysis. Two hundred and twenty-one F1 plants using the *ubc22* mutant as the maternal parent and one hundred F1 plants using the WT as the maternal parent were analyzed. In the floral bud tissues, there are two peaks, representing 2C and 4C nuclei (see Figure 5). For comparative analysis among different individuals, the relative value of the 4C peak was used. Parameters of the flow cytometry were set so that the average WT 4C peak value is about 50. The relative 4C values are grouped with increments of 5 s. The 4C peaks of WT samples were around 50 in the group of 47.5–52.5, while the 4C peak values of the plants with a nuclear DNA content of the triploid were in the group of 72.5–77.5.

To assess the frequency of abnormal gametes in the mutant, we analyzed F1 plants produced from reciprocal crosses between the WT and a *ubc22* mutant. An analysis of 100 F1 plants from the cross using the WT as the maternal parent showed that 99% of the plants were normal diploids, and only one plant had a nuclear content that was slightly less than diploid (Figure 6). The one plant with a nuclear DNA content less than diploid could be due either to the abnormal gamete or to a slight sample variation in nuclear staining. In any case, the results indicate that the male gametes are mostly normal in terms of nuclear DNA content and that abnormal gametes are very rare. On the other hand, an analysis of 219 F1 plants using the *ubc22* mutant as the maternal parent showed that approximately 75.8% of them were diploids, 3.7% were triploids, 20.5% were aneuploids and 2.3% had a DNA content less than the WT diploids (Figure 6). It needs to be noted that although 3.7% of the plants had a DNA content similar to that of the triploid, we do not know whether they were true triploids with three complete sets of chromosomes; they could be aneuploids with a DNA content similar to a true triploid. Considering that the uneven segregation of chromosomes was frequently observed, while neither of the two meiotic cell divisions were completely arrested in the mutant, most of the mutant plants with abnormal DNA contents would be expected to be aneuploids. Overall, these results suggest that about 24% of the female gametes which could be fertilized and develop into seeds have abnormalities in terms of their nuclear DNA content.

### 2.5. Analysis of Nuclear DNA Content in ubc22 Mutant Plants

**Figure 7:**
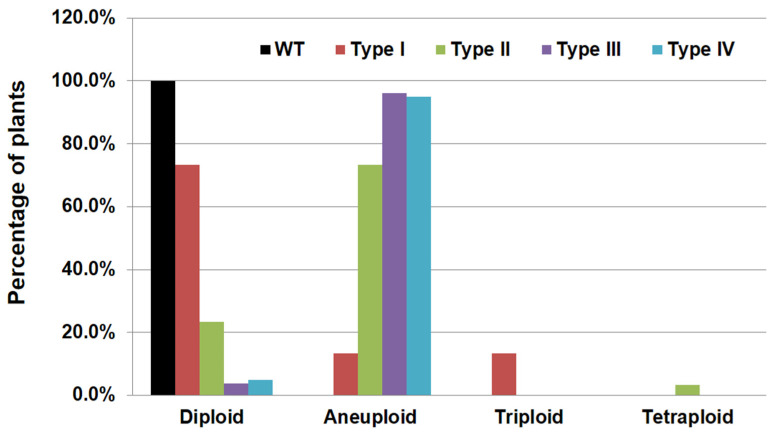
Ploidy analysis of different subtypes of *ubc22-1* mutant. Forty-five type I, thirty type II, twenty-six type III and twenty type IV plants were analyzed. Leaf tissues of four-week-old plants were used, and nuclei were stained with DAPI. Thirty-eight WT (diploid) plants were used as references. Based on the nuclear DNA contents (4C peak values), the plants were sorted into the groups of diploids, aneuploids, triploids and tetraploids.

It has been observed that the *ubc22* mutant plants display a wide range of phenotypes, and could therefore be grouped into four subtypes based on rosette leaf morphology as well as plant size: type I—plants similar to the WT; type II—plants with rounder leaves; type III—plants with relatively long pale leaves (a higher length/width ratio); and type IV—plants with small and narrow serrated leaves [45]. The observation of chromosome abnormalities and changes in the nuclear DNA content raise the question as to whether different subtypes have different characteristics of ploidy changes. Therefore, we analyzed the relative DNA content of different subtypes using about 30–40 four-week-old plants for each subtype. Under the flow cytometric settings, all of the 38 WT plants had a 4C peak value close to 50, which served as a reference for the mutant plants (Figure 7).

For type I plants, which are similar to the WT, 73.3% were diploids, while there was about 13.3% of both triploid and aneuploid plants (Figure 7). For type II mutant plants, 23.3% were diploids, 73.3% were aneuploids and 3.3% (one out of thirty) was a tetraploid. For type III and IV plants, the vast majority of them (96.2% and 95%, respectively) were aneuploids and only 3.8% and 5% of them were diploids (Figure 7). Those results indicate that type I plants were mostly diploids, that type II plants were unique with a relatively high percentage (13.3%) of triploids and that very high percentages (over 95%) of type III and type IV plants were aneuploids.

## 3. Discussion

### 3.1. UBC22 Is Critical for Female Meiosis, but Not for Male Meiosis

Little is known regarding the roles of plant E2 enzymes in meiosis. The involvement of several E2 enzymes in metazoan and yeast meiosis has been reported. In mouse oocytes, Ube2C, Ube2S and Ube2D3 are necessary for the first meiotic division through the regulation of APC/C activity and the degradation of securin before anaphase I [50]. Ube2C (also referred to as UbcH10) initiates the formation of ubiquitin chains and Ube2S elongates K11-linked ubiquitin chains [51,52], while Ube2D3 (also known as UbcH5C) acts on a separate path by interacting with the APC/C to ubiquinate securin for its degradation [50]. In budding yeast, Ubc13 also plays a critical role in initiating meiotic recombination [53]. In this study, results based on several different analyses, including of DMC1 distribution, the deposition pattern of callose, chromosomal behavior during meiosis and the ploidy level of mutant plants clearly indicate that Arabidopsis UBC22 is critical for female meiosis. The uneven segregation of chromosomes in meiosis I was often observed in female meiosis I. However, only a very small number of ovules analyzed were at the suitable stages and would allow for the counting of chromosomes following chromosome spreading, making the accurate counting of MMCs with abnormal chromosome segregation difficult. The results from analyzing over 200 F1 plants using the *ubc22* mutant as the female parent revealed clearly that about 24% of female gametes had an abnormal nuclear DNA content, consistent with the chromosome spreading observations. It should be noted that the estimate of 24% of female gametes having an abnormal nuclear DNA content was based on the F1 plants, which were produced from the successful fertilization of mutant female gametes by WT pollen. The estimate, however, does not include mutant female gametes which were defective and unable to produce F1 plants. It has been observed that, in *ubc22* mutants, over 70% of ovules did not have an FM or had an abnormal FM; furthermore the number of seeds per silique was reduced by almost 90% [44], suggesting that the percentage of abnormal female gametes is likely much higher. Since UBC22 represents a type of plant E2 enzyme able to catalyze K11-linked ubiquitination [44], the present results thus have identified one important function of this plant E2 enzyme type in female meiosis.

Several genes and their encoded proteins with possible regulatory functions in plant meiosis have been identified mostly from Arabidopsis, including AtSPO11-1 (a homologue of the yeast Spo11 that catalyzes DSBs) [19], SYN1 (homolog of REC8, a subunit of the meiotic cohesin complex) [54], ASY1 (required for the synapsis of homologous chromosomes) [4] and its rice ortholog PAIR2 [5], DYAD/SWI1 (needed for the loading of sister chromatid cohesion) [55] as well as Action-Related Protein 6 (ARP6, a subunit of the SWR1 ATP-dependent chromatin-remodeling complex that functions in meiotic recombination) [56]. Mutations of these genes have severe consequences on plant meiosis. Most of the analyses were performed using male meiocytes (pollen mother cells) due to the ease of sample access and isolation, but defects in both male and female meiosis have been reported in the mutants of AtSPO11-1 [19], SYN1 [57] and its rice homolog PAIR2 [5], DYAD/SWI1 [55] and ARP6 [56,58]. These results indicate that many regulatory factors are shared by both male and female meiosis in plants.

There has been very limited information regarding the differences in the regulatory aspects between plant male and female meiosis. Few genes have been identified to be specifically important for plant female meiosis. In *ubc22* mutants, abnormalities in chromosome segregation and gamete ploidy were observed for female meiosis, but not for male meiosis. These results have thus identified a major difference between female and male meiosis in the requirement for UBC22-mediated ubiquitination. Previously, based on DIC microscopic observations, it was suggested that the degeneration of the FM in the *ubc22* mutant is likely due to programmed cell death instead of the inhibition of meiosis [44]. Results from this study further show that while female meiosis in the mutant is initiated, chromosome segregation is abnormal, revealing an important role of UBC22 in maintaining genome stability. Further, UBC22 provides an interesting lead for studying the regulation that is specific to plant female meiosis.

### 3.2. ubc22 Mutant Plants Have a Frequent Occurrence of Chromosome Abnormalities

The abnormal chromosome segregation indicates that female meiosis I in *ubc22* mutants is affected. The abnormal chromosomal segregation is likely responsible for the high levels of aneuploids in the *ubc22* mutant plants and the functional megaspore abnormalities in the majority of *ubc22* ovules, as reported previously [44]. It will be interesting to determine what causes the abnormal chromosome segregation. The meiotic recombinase DMC1 localizes to the meiotic DSB sites and promotes interhomolog DNA repair of meiotic DSBs (Kurzbauer et al., 2012). The mutation of DMC1 results in interrupted meiotic DSB repair, failure to form a normal synaptonemal complex, and 100% univalents (Bishop et al., 1992). DMC1 distribution and deposition at meiotic DSBs in MMCs are thus important for normal chromosome segregation during female meiosis. The more diffused distribution and less strong foci of DMC1 suggest that the loading of DMC1 at meiotic DSBs might be affected in the MMCs of *ubc22* mutants. Considering that UBC22 is an E2 enzyme, it will be interesting to know the specific protein responsible for the reduced loading of DMC1 onto the foci as well as the possible impact of reduced DMC1 loading on the repair of DSBs in *ubc22* female meiosis. On the other hand, and interestingly, homologous chromosomes appeared as bivalent chromosomes in female meiosis I in the mutant ovule, and the chromosome axis-associated protein ASY1 showed a distribution similar to that in the WT. Thus, chromosome segregation is severely affected in female prophase I of *ubc22* mutants, while the pairing of the homologous chromosomes appears normal or not significantly affected. 

About 70% of the *ubc22* mutant plants show normal leaf morphology and vegetative growth compared to the WT, while about 30% of the mutants could be grouped into three other subtypes based on leaf morphology and size [45]. The high frequencies of aneuploids and polyploids observed raises the question as to whether aneuploids and polyploids may be the cause for the distinct phenotypes of some subtypes. The results from the ploidy analysis of the four subtypes of mutant plants show that aneuploids and polyploids were found in much higher frequencies in the three subtypes with stronger and more distinct mutant phenotypes. Among the type I plants (the subtype with fairly normal leaf morphology and size), about 73% were diploids while 27% were aneuploids and polyploids. For type II plants, about 77% of them were aneuploids and polyploids. For type III and type IV plants (more severe phenotypes), over 95% were aneuploids. Thus, the high frequencies of chromosome abnormalities and genetic imbalance could be a major factor for the distinct phenotypes of type II, type III and type IV mutant plants.

Interestingly, one mutant plant among over 110 plants analyzed had the DNA content of a tetraploid (Figure 7). Since the male gametes of the *ubc22* mutants are normal, this result indicates that the female gamete used to produce the plant likely had a nuclear DNA content equivalent to a triploid, although the frequency was very low. If the chromosomes or chromatids fail in segregation and all end up in one daughter cell in one meiotic division while the other meiotic division proceeds normally, only diploid gametes would be produced. The existence of a plant with a nuclear DNA content of the tetraploid suggests that chromosomes and chromatids in both meiotic divisions could segregate abnormally in *ubc22* mutant MMCs. Consistent with this suggestion, among 219 F1 plants using a mutant as the female parent, a small number of plants had a DNA content higher than the triploid (Figure 6), suggesting that female gametes for producing those F1 plants had a DNA content higher than a diploid. 

### 3.3. Function of UBC22 in Female Meiosis

In humans, Ube2S works with the APC/C to elongate K11-linked polyubiquitin chains on APC/C substrates, following the initial priming ubiquitination of the substrates by another E2 enzyme (Ube2C/UbcH10 or Ube2D/UbcH5) with the APC/C [51,52]. It has also been shown that Ube2S and Ube2C are critical for regulating the progression of female meiosis [50,59]. The chromosomal abnormalities and sequence similarity of UBC22 to human Ube2S and animal homologs point to the possibility that UBC22 may regulate female meiosis through the APC/C.

The APC/C is a large complex in eukaryotes, consisting of 14 or more core subunits in vertebrates, yeast and plants, as well as additional co-activators [36,60]. Regarding the plant APC/C core subunits, studies using Arabidopsis T-DNA knockout mutants have shown that homozygous mutants could not be obtained, indicating the essentiality of the APC/C [61,62,63,64,65,66], while the heterozygous mutants of APC1, APC2, APC4 and APC6 display strong defects in female gametogenesis [61,62,63,64]. In addition, the mutant of APC11 has a zygote-arrest phenotype [66]. Interestingly, a mutant with a point mutation in APC8 has defects in male meiosis but not in female meiosis [37]. These results show that APC/C has a range of important functions. However, little information is available on the role of APC/C in female meiosis based on these heterozygous mutants. 

Thus, among the mutants of plant APC/C subunits, none appear to resemble *ubc22* mutants, suggesting that UBC22 is particularly important for female meiosis and megasporogenesis. Based on the function of UBC22 as an E2 enzyme, it may be inferred that the degradation of certain substrate proteins is deregulated during female meiosis in the *ubc22* mutant, resulting in the abnormal chromosome segregation. It will be important to identify the E3 ligase component that works with UBC22 and the substrates in order to gain more mechanistic understanding on the function of K11-linked ubiquitination as well as the differences between female and male meiosis in plants.

## 4. Materials and Methods

### 4.1. Plant Growth

*Arabidopsis thaliana* plants were grown in 4-inch square pots and placed in a growth chamber or a growth room (20 °C constant, 16/8 h day/night photoperiod with a daylight fluence rate of 90–120 µm/m^2^/min). 

### 4.2. Aniline Blue Staining of Callose

Young gynoecia (0.5 to 1 mm in length) were collected, prepared, fixed and stained in 0.1% aniline blue as described in [47]. Ovules were picked up and mounted on a glass slide. Fluorescence was observed under a Leica DM2500 microscope using a 340–380 nm bandpass excitation filter and a 425 nm longpass emission filter.

### 4.3. Whole-Mount Immunolocalization of DMC1 Protein

Young gynoecia were collected and processed for the whole-mount immunolocalization of DMC1 in the ovule, as described in [67]. A rabbit anti-DMC1 antibody [68] was used at a 1:100 dilution, and an Alexa-Fluor-488–labeled goat anti-rabbit secondary antibody was used at a 1:500 dilution. After counterstaining with propidium iodide (PI), the ovules were examined and images were captured using a Leica SP5 confocal microscope (488 nm laser and emission bandpass of 500–550 nm for the detection of Alexa Fluor 488; 568 nm laser and emission bandpass of 575–615 nm for the detection of PI). 

### 4.4. Chromosome Spread

Young gynoecia (0.5 to 1 mm in length) were dissected into small strips, fixed in FAA solution (formaldehyde: acetic acid: ethanol: water (2:1:10:7)) for 2 h, and washed sequentially in 50% ethanol, 25% ethanol, 15% ethanol, water and 10 mM of citrate acid for 5 min for each step. They were digested with 0.2% Macrozyme R10 and 0.2% Cellulase R10 (both were from Research Products International RPI, Mt Prospect, IL, USA) at 37 °C for one hour. Following the digestion, the samples were washed with 10 mM of citrate acid once for 5 min and then transferred into one drop of 60% acetic acid on a glass slide which was placed on a 45 °C hotplate for 1 min. The samples were rinsed with FAA for 2 min, and excess FAA solution was drained away before the samples were stained with 2 μg/mL DAPI (4′,6-diamidino-2-phenylindole) for 10 min. For cell wall staining, the samples were incubated with 10 μL of 0.2% Calcofluor White and 10 μL of 10% KOH for 1 min, washed twice with ddH2O and mounted in the mounting solution (2% n-propyl gallate and 25% glycerol in PBS). The samples were covered with a coverslip and observed under an LSM microscope with 405 nm laser excitation.

### 4.5. Analysis of Nuclear DNA Content by Flow Cytometry

The young buds or young leaves of four-week-old plants were used for nuclear DNA content analysis. The samples were chopped with a sharp blade in 500 µL of a nuclei isolation buffer (10 mM of MgSO_4_, 50 mM of KCL and 5 mM of HEPES with the pH adjusted to 8.0), followed by the addition of 500 μl of a nuclear staining solution (nuclei isolation buffer with 1.5 μg/mL of DAPI). The samples were filtered through a 30 μm filter and left for a couple of minutes before being analyzed with a Partec Ploidy Analyser (Partech). Typically, about 2000 nuclei were measured in each run.

## Figures and Tables

**Table 1:** Callose formation in the early stages of megasporogenesis in WT and *ubc22-1* mutant ovules.

Line	Ovules Observed	Callose Staining along Meiocyte Cell Wall (Type 1)	Clear Callose Band (s) (Type 2)	Callose Band with Staining in Other Places (Type 3)	Disorganized Callose Deposition and No Clear Band (Type 4)
WT	117 (100%)	30 (25.6%)	87 (74.4%)	0	0
*ubc22-1*	103 (100%)	36 (35.0%)	11 (10.7%)	18 (17.5%)	38 (36.9%)

Flowering buds (the sixth bud counting from the newly opened flower on an inflorescence stem) were dissected, stained, prepared and then observed under a microscope equipped with DIC optics. Ovules in an early stage of meiotic division (with the inner integument below half the length of the nucellus) were included in the analysis. Four types of ovules were counted: Type 1—weak callose staining observed along the cell wall of an MMC before meiosis. Type 2—one or two clear callose bands observed during meiosis of an ovule. Type 3—one or two callose bands were recognizable, but a diffused callose signal was observed in other locations as well. Type 4—callose deposition was disorganized, and no clear callose bands were observed. A large portion of the mutant ovules had diffused or disorganized callose deposition compared to the WT ovules that showed one or two clear bands.

## Data Availability

All data have been provided in the manuscript as main figures and tables or as supplementary materials.

## Supplementary Materials

The following are available online at https://www.mdpi.com/article/10.3390/plants10112418/s1, Figure S1. Presence of callose in the late stages of megasporogenesis in WT and *ubc22-1* mutant ovules. Figure S2. DMC1 immunolocalization in representative WT and *ubc22* mutant ovules. Figure S3. ASY1 immunolocalization in WT and *ubc22* mutant ovules. Table S1. Meiosis in male meioctyes of WT and *ubc22* mutant plants.
