# Supplementary material for: The Inactivation of Arabidopsis UBC22 Results in Abnormal Chromosome Segregation in Female Meiosis, but Not in Male Meiosis
Source: Plants (Basel). 2021 Nov 9;10(11):2418. doi: 10.3390/plants10112418 (PMC8625819; doi:10.3390/plants10112418)
Supplement: Supplementary file 1 [file plants-10-02418-s001.zip › plants-1360007-supplementary.pdf]

Supplementary Materials

Table S1. Presence of callose at late stages of megasporogenesis in WT and *ubc22-1* mutant ovules.

Line	Ovules observed	fluorescence signal as two bands (Type 1)	fluorescence signal as a line or a spot at the micropylar end (Type 2)	No fluorescence signal (Type 3)	Strongly disorganized signal (Type 4)
WT	126 (100%)	2 (1.6%)	65 (51.6%)	59 (46.8%)	
ubc22-1	131 (100%)	1 (0.8%)	39 (29.8%)	9 (6.9%)	82 (62.6%)

Floral buds (the fourth bud from the newly opened flower) were dissected, stained, prepared and then observed under a microscope equipped with DIC optics. Ovules at the functional megaspore (FM) stage (with the inner integument close or just below the top of nucellus) were included in the analysis. Four types of callose staining pattern were observed. Type 1: the presence of two callose bands near the micropylar end. Type 2: one callose band or spot at the micropylar end. Type 3: no callose staining observed. Type 4: strongly disorganized callose staining. A large portion of mutant ovules had strongly diffused or disorganized callose staining compared to WT ovules which had weak or no callose staining.

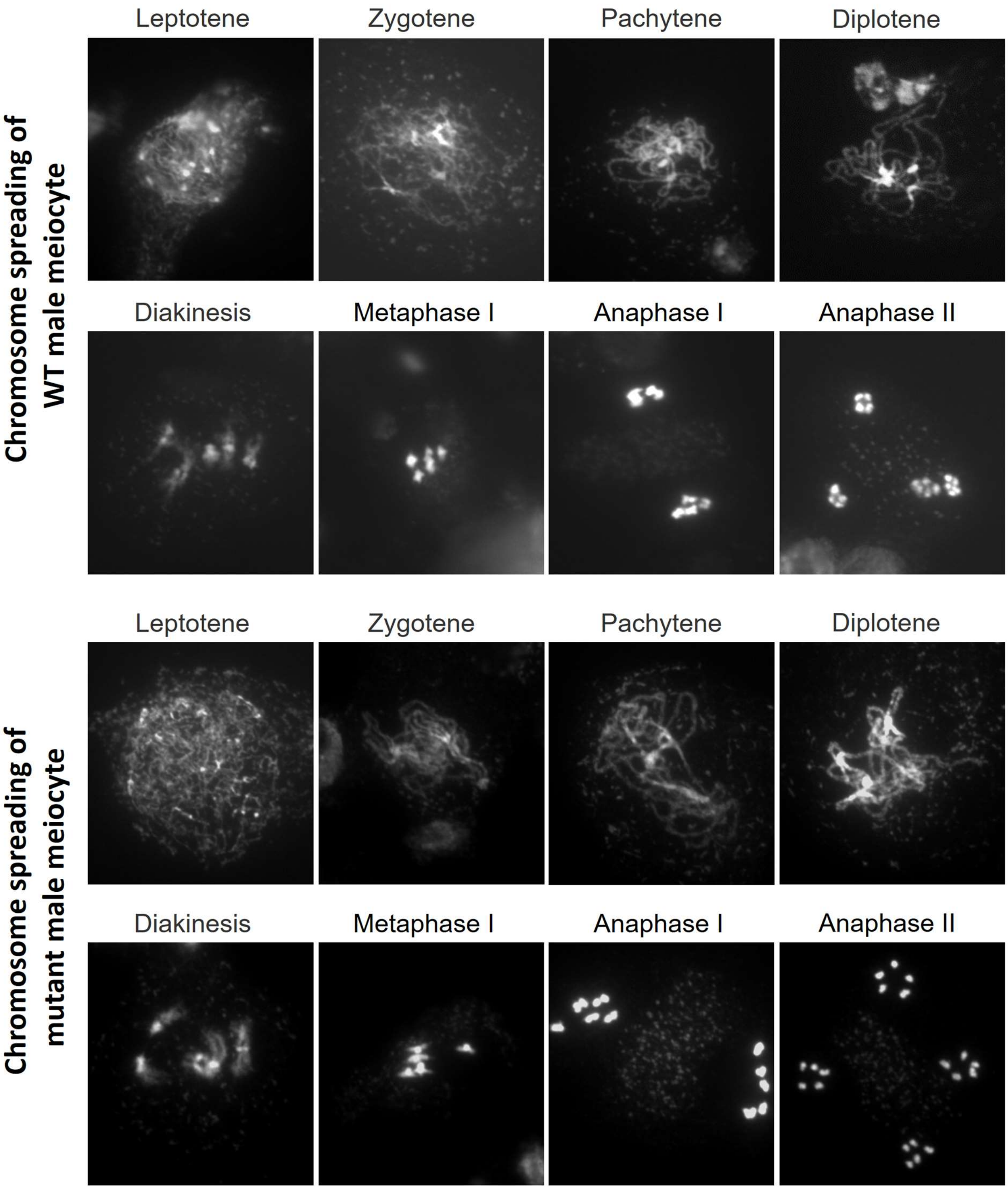

Figure S1. Meiosis in male meioctyes of WT and *ubc22* mutant plants.

A

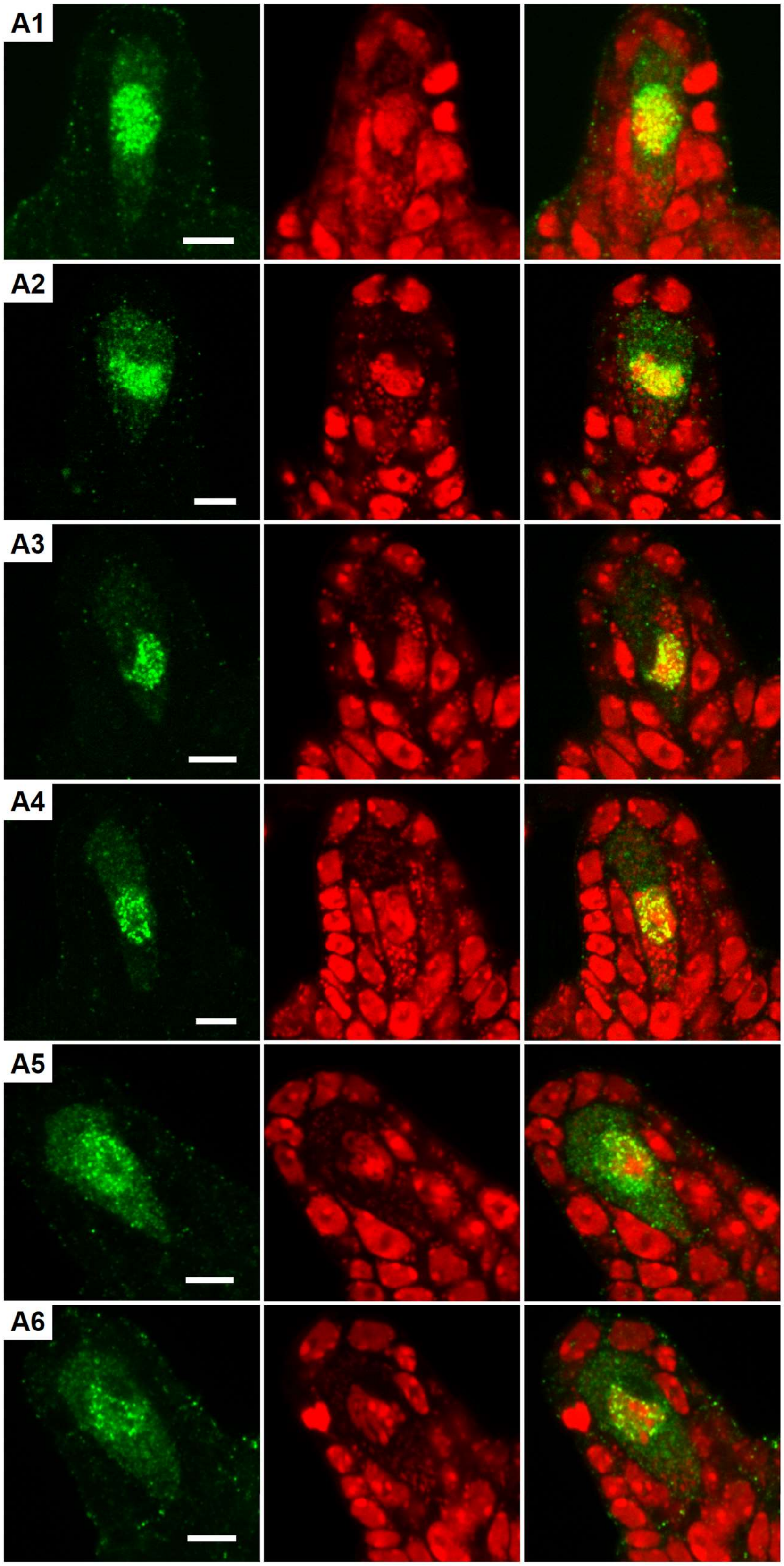

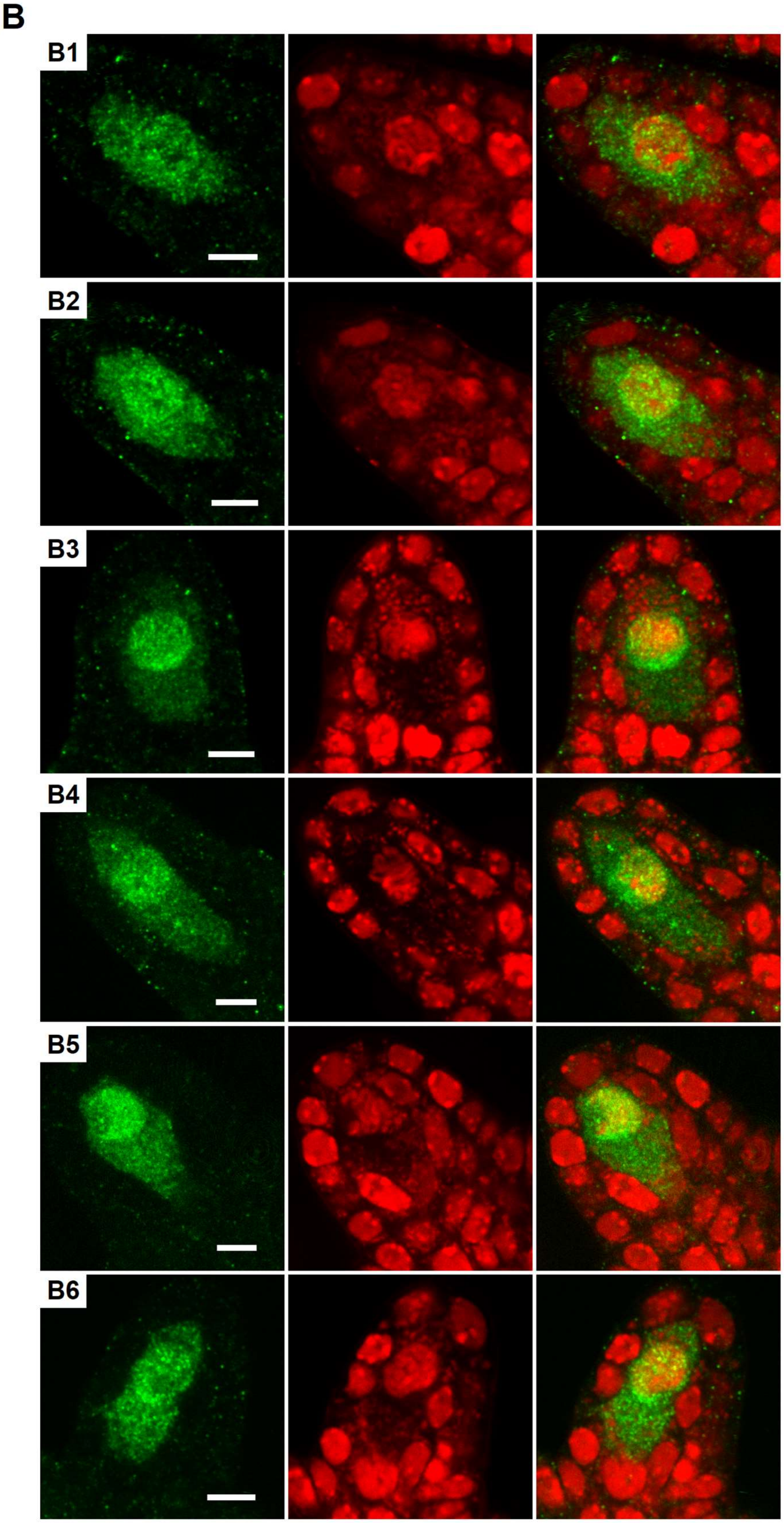

Figure S2. DMC1 immunolocalization in representative WT and *ubc22* mutant ovules. WT and

ubc22-1 mutant ovules were dissected, fixed and processed for immunostaining with an antibody against DMC1 (green color). They were also stained with propidium iodide (PI, red color). Six WT and six mutant ovules are shown. (**A**) In the WT ovules, DMC1 accumulated during prophase I in the MMC as clear and strong foci. (**B**) In majority of *ubc22-1* ovules, DMC1 signal showed a more diffused distribution with less strong foci. In each row, the left image shows DMC1 staining, the center shows PI staining and the right shows the overlay of the two images. Scale bars = 5 μm.

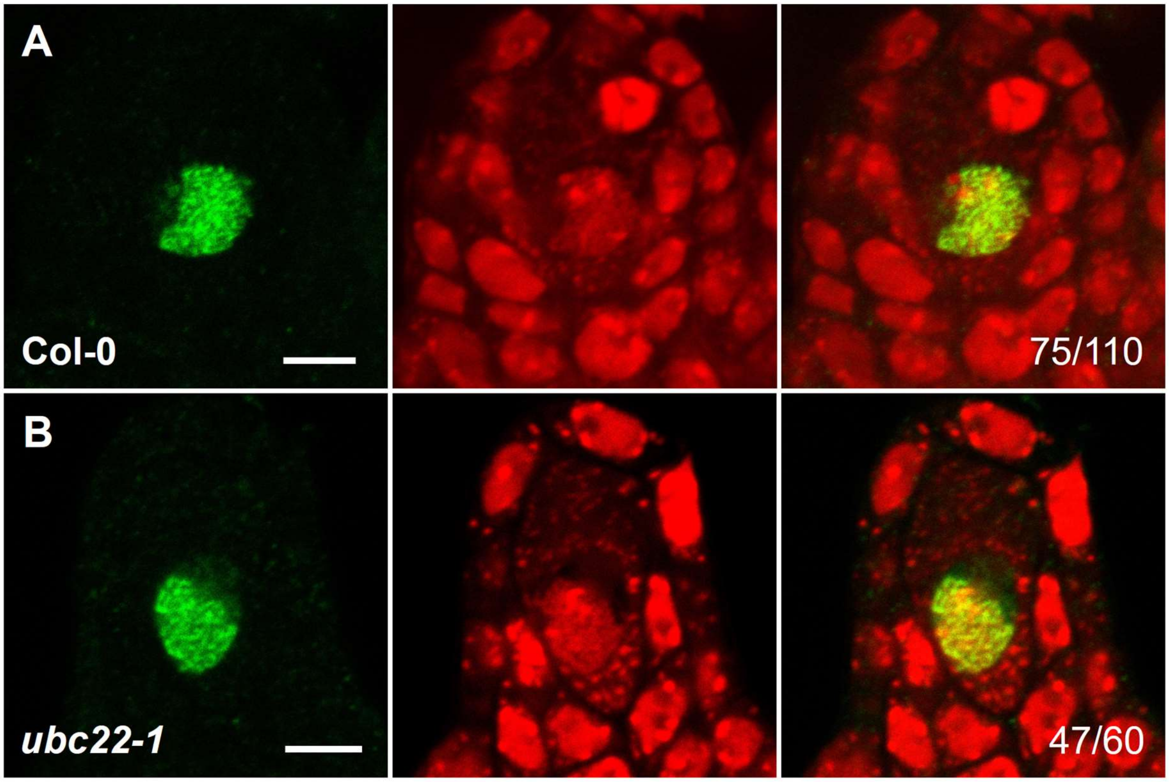

Figure S3. ASY1 immunolocalization in WT and *ubc22* mutant ovules. WT and *ubc22-1* mutant ovules were dissected, fixed and processed for immunostaining with an antibody against ASY1 (green color). They were also stained with propidium iodide (PI, red color). (**A**) One WT ovule. (**B**) One *ubc22-1* ovule. In each row, the left image shows ASY1 staining, the center shows PI staining and the right shows the overlay of the two images. The numbers in the right images indicate the number of the ovules with the expression pattern among the total number of ovules examined. Scale bars = 5 μm.
